# Association of Prognostic Nutritional Index and New-Onset Atrial Fibrillation in Patients Undergoing Surgical Aortic Valve Replacement: A Silent Predictor in Perioperative Outcomes?

**DOI:** 10.3390/jcm15020555

**Published:** 2026-01-09

**Authors:** Cecilia Vecoli, Augusto Esposito, Ludovica Simonini, Valentina Zanetti, Maria Serena Parri, Luca Bastiani, Pier Andrea Farneti, Ilenia Foffa

**Affiliations:** 1 Institute of Clinical Physiology, CNR, 54100 Massa, Italy; cecilia.vecoli@cnr.it (C.V.); ludovicasimonini@cnr.it (L.S.); luca.bastiani@cnr.it (L.B.); ilenia.foffa@cnr.it (I.F.); 2 Fondazione Toscana Gabriele Monasterio, 54100 Massa, Italy; vzanetti@ftgm.it (V.Z.); parri@ftgm.it (M.S.P.); farneti@ftgm.it (P.A.F.)

**Keywords:** new-onset postoperative atrial fibrillation (NOAF), surgical aortic valve replacement, prognostic nutritional index (PNI)

## Abstract

**Background**: New-onset postoperative atrial fibrillation (NOAF) is the most prevalent arrythmia after cardiac surgery with a significant clinical and economic impact. Therefore, simple and practical biomarkers for NOAF prediction remain a clinical priority. Increasing evidence indicates that malnutrition is linked to postoperative complications, including the onset of atrial fibrillation. The Prognostic Nutritional Index (PNI), which reflects the immunonutritional and inflammatory status through serum albumin concentration and lymphocyte count, has emerged as a reliable prognostic indicator in cardiovascular disease. The present study aimed to investigate the association between PNI and the development of NOAF in patients undergoing surgical aortic valve replacement (SAVR). **Methods**: A total of 241 consecutive patients who underwent AVR for severe aortic stenosis or regurgitation were enrolled in this study. The population was stratified into two groups according to the development of NOAF (NOAF group) or the lack thereof (no NOAF group). **Results**: In both univariate and multivariate logistic regression analyses adjusted for several established NOAF determinants, age and PNI, both as continuous variables, were independently associated with NOAF in both univariate (OR = 1.03; CI 95% = 1.01–1.06, *p* = 0.009, and OR = 0.9; CI 95% = 0.8–0.9, *p* = 0.01, respectively) and multivariate models (OR = 1.02; CI 95% = 1.01–1.06, *p* = 0.05, and OR = 0.9; CI 95% = 0.8–0.9, *p* = 0.03, respectively). When PNI was analyzed by tertiles, patients in the lowest tertile (PNI < 41.5) showed a significantly higher risk of developing NOAF at both univariate (OR = 1.9; CI 95% = 1.2–2.8, *p* = 0.004) and multivariate analysis (OR = 1.6; CI 95% = 1–2.6, *p* = 0.03), whereas age lost statistical significance (OR = 1.0; 95% CI = 0.9–1.05; *p* = 0.06). Furthermore, when the study population was divided into two groups based on the median age (70 years), PNI values differed significantly between NOAF and no NOAF patients only in patients under 70 years (*p* = 0.01). In this younger subgroup, PNI remained an independent predictor of NOAF, both when considered as a continuous variable (OR = 0.86; CI 95% = 0.74–0.98, *p* = 0.02), and nominal variable (PNI < 41.5, OR = 0.88; CI 95% = 0.80–0.97, *p* = 0.01). **Conclusions:** Overall, these findings identify PNI as an independent predictor of NOAF following SAVR, particularly in patients younger than 70 years. This study underlines the potential clinical value of preoperative nutritional assessment for risk stratification. Incorporating nutritional parameters such as PNI into current predictive models may enhance the accuracy of prognostic evaluation and support targeted perioperative management strategies.

## 1. Introduction

New-onset postoperative atrial fibrillation (NOAF) represents the most frequent arrhythmia following cardiac surgery, with an incidence ranging from 31% to 64%. Cohort studies have reported an approximate incidence of 30% among patients undergoing isolated aortic surgery [1], with even higher rates observed in combined valve and coronary artery bypass graft (CABG) procedures [2,3,4,5,6,7]. Despite its higher frequency, the pathogenesis of NOAF is incompletely understood. Current evidence supports a multifactorial aetiology involving the interaction of pre-existing structural and electrophysiological abnormalities with perioperative inflammatory and metabolic factors. However, simple and clinically practical biomarkers capable of reliably predicting NOAF are still lacking.

NOAF is not a benign entity because its occurrence is associated with increased morbidity, prolonged hospitalization and risk of all-cause mortality. Moreover, the need for long-term anticoagulation is associated with risks of bleeding and stroke [4,8]. Owing to the considerable clinical and economic consequences of postoperative atrial fibrillation, numerous investigations have sought to identify risk factors to guide targeted preventive strategies [9,10,11]. Identifying modifiable risk factors, in particular, is essential to reduce the incidence of NOAF and its impact on both patient outcomes and healthcare systems.

Among the established predictors of new-onset atrial fibrillation, advanced age consistently emerges as the strongest and most reliable independent determinant. Epidemiological evidence indicates a marked age-related increase in NOAF incidence, with patients aged ≥ 72 years exhibiting an approximately fivefold higher risk compared with those aged ≤ 55 years [12,13]. This pronounced association likely reflects age-related structural and electrical remodeling of the atria, as well as the cumulative burden of comorbidities and systemic inflammation commonly observed in older individuals.

In contrast, the influence of sex on the risk of NOAF remains controversial. Several studies have reported that male sex is associated with a higher incidence of NOAF [14,15], potentially due to differences in atrial size, hormonal profiles, and cardiovascular risk burden. However, other investigations have failed to confirm a significant association between sex and NOAF risk, suggesting that this relationship may be modulated by additional clinical or procedural factors [13,16].

Beyond age and sex, multiple clinical risk factors for NOAF have been identified. These include a prior history of atrial fibrillation, increased left atrial size, mitral valve disease, previous cardiac surgery, chronic obstructive pulmonary disease (COPD), white race, and physical inactivity [17,18,19]. Collectively, these factors contribute to atrial structural remodeling, electrical instability, and autonomic imbalance, thereby increasing susceptibility to post-operative arrhythmias. Moreover, among perioperative risk factors of NOAF, inflammation status seems to have a strong relation with the development of NOAF [20]. An exaggerated inflammatory response following cardiac surgery may promote atrial fibrosis, oxidative stress, and electrophysiological alterations, ultimately facilitating the onset of NOAF.

In recent years, growing attention has been directed toward the role of nutritional status in cardiovascular health and clinical outcomes. Previous investigations have consistently established a relationship between malnutrition and adverse clinical outcomes, including prolonged length of hospital stay, increased mortality, higher readmission rates, and elevated healthcare costs, underscoring the importance of early nutritional assessment and intervention [21,22,23]. Furthermore, several studies have highlighted the impact of nutritional status on the development of atrial fibrillation [24,25,26,27]. Most research in this field has primarily focused on the relationship between obesity and AF, showing that obese patients have a 12% higher risk of developing NOAF compared with non-obese individuals [28]. In contrast, the potential role of malnutrition has been relatively underexplored. This represents a significant gap in the literature, particularly considering that malnutrition is highly prevalent among hospitalized patients. Indeed, recent data from the United States and Europe indicate that nearly one-third of inpatients are malnourished or at risk of malnutrition at the time of hospital admission [29,30,31].

Only a limited number of studies have explored the influence of malnutrition on new-onset atrial fibrillation, leaving the association poorly defined [32,33,34,35].

The Prognostic Nutritional Index (PNI), calculated from serum albumin concentration and lymphocyte count, has been widely adopted to evaluate immune-nutritional and inflammatory status and to predict prognosis in various malignancies and systemic diseases [36,37]. Because PNI reflects both malnutrition and systemic inflammation, it has also been associated with poor outcomes in multiple cardiovascular conditions [38,39]. Additionally, a strong association between PNI and atrial fibrillation has been found [36,40].

In this paper, we aimed to investigate the possible role of PNI in predicting NOAF in patients undergoing surgical aortic valve replacement (SAVR).

## 2. Methods

### 2.1. Study Design and Patients

This is a prospective, observational, and single-center study conducted at Fondazione Toscana G. Monasterio. Between July 2024 and March 2025, 241 consecutive patients were enrolled to undergo aortic valve replacement (AVR) for severe stenosis or insufficiency, with or without an associated thoracic aortic aneurysm. All patients underwent routine evaluation before surgery. The evaluation encompassed medical history review, physical examination, electrocardiography, laboratory testing, and echocardiography conducted by cardiologists with expertise in cardiac imaging, in addition to a preoperative anaesthetic assessment. This study received ethical approval by the local Ethics Committee (Comitato Etico di Area Vasta Nord Ovest [CEAVNO] Study ID No. 26429/2024). All patients included in this study signed a written informed consent.

For all patients, the severity of aortic valve dysfunction, the type of surgical approach (full sternotomy, hemi-sternotomy, or mini-thoracotomy), and any concomitant procedures—including aortic surgery, mitral valve surgery, coronary artery bypass grafting (CABG), or left atrial appendage closure (LAAC) for atrial fibrillation—were recorded. Hospital length of stay was calculated for each patient from the day of the valvular procedure to discharge. All complications during the hospitalization were recorded. NOAF was described as an AF episode ≥ 30 s during hospitalization confirmed immediately post episode by 12-lead ECG. All patients underwent continuous cardiac telemetry starting 24 h before surgery and continuing until hospital discharge, which typically occurred 5–7 days postoperatively in the absence of major complications. Patients with a history of atrial fibrillation, regardless of prior antiarrhythmic drug use, were excluded. In our hospital, patients who developed atrial fibrillation underwent a standard of care prophylaxis that consist initially with pharmacological cardioversion, most commonly using amiodarone; if unsuccessful, electrical cardioversion was subsequently performed, followed by rate control therapy. The PNI as well as all the other inflammatory-nutritional indexes including Neutrophil-to-lymphocyte ratio (NLR), Systemic immune inflammation index (SII), Systemic Inflammatory Response Index (SIRI), Inflammatory prognosis index (IPI), Blood urea nitrogen to albumin ratio (BAR) were calculated from biochemical parameters measured prior to the surgical intervention, as previously described [40,41,42,43].

### 2.2. Statistical Analysis

Continuous variables are reported as mean ± standard deviation (SD), whereas categorical variables are expressed as relative percentages. Continuous variables were compared using Student’s *t*-test or the Mann–Whitney U test, depending on whether the data were normally distributed. Categorical variables were compared using the chi-square test. Logistic regression analysis was conducted to evaluate the association between individual covariates, pre-selected based on clinical relevance, and the risk of developing NOAF. The study population was stratified into two groups according to the median age (70 years) while PNI was stratified into tertiles (low tertile PNI < 41.5, medium tertile 41.6 < PNI < 45.0, and high tertile PNI > 45.1). All statistical analyses were performed using Stata/SE 13.1 and SPSS Version 24. A *p*-value < 0.05 was considered statistically significant.

## 3. Results

The overall baseline characteristics of the study population are presented in Table 1.

The mean age of the study population was 66.2 ± 11.5 years, and 63.9% of patients were males. Bicuspid aortic valve was identified during surgery in 42.3% of patients. A total of 138 patients (57.3%) underwent isolated SAVR, while the remaining patients underwent SAVR combined with other procedures such as aorta surgery, mitral valve surgery, CABG, and left atrial appendage closure (in 21.2%, 5%, 7.1% patients, 1.2%, respectively). Full sternotomy was performed in 28.6% of patients, and mini thoracotomy in 19.9%, while mini sternotomy was achieved in 51.5% of patients. Regarding the post-operative atrial fibrillation, eighty-four patients (36.8%) developed NOAF.

In Table 2, the population was stratified according to the development of NOAF (NOAF group) or the lack thereof (no NOAF group).

Regarding clinical characteristics, no significant differences were observed between the NOAF and no NOAF groups, except for age. Patients who developed NOAF also experienced a longer hospital stay (*p* = 0.009). There were no statistically significant differences between groups in the prevalence of cardiovascular risk factors, treatments, surgical data, or preoperative echocardiographic parameters. In contrast, biochemical analyses revealed that albumin levels (*p* = 0.01) and the PNI (*p* = 0.01) were significantly different between the NOAF and no NOAF groups. Univariate logistic regression analyses identified age (OR = 1.03; CI 95% = 1.01–1.06, *p* = 0.009) and PNI (OR = 0.9; CI 95% = 0.8–0.9, *p* = 0.01) as independent variables associated with the development of NOAF. A multivariate logistic regression analyses adjusted for several established NOAF determinants as Left atrial volume indexed, ejection fraction (EF), surgical complexity and coronary artery bypass graft concomitant established age and PNI, as continuous variables, to be two parameters independently associated with NOAF (Table 3).

Interestingly, when PNI was stratified into tertiles, the lowest tertile (PNI < 41.5) was the only independent risk factor for NOAF at univariate (OR = 1.9; CI 95% = 1.2–2.8, *p* = 0.004) and multivariate analysis (OR = 1.6; CI 95% = 1–2.6, *p* = 0.03), whereas age lost statistical significance (OR = 1.0; 95% CI = 0.9–1.05; *p* = 0.06).

When the study population was divided into two groups according to the median age (70 years), PNI index was significantly different between NOAF and no NOAF group only in patients under 70 years (*p* = 0.01). Moreover, only in this population PNI, as continuous and nominal variable, was an independent variables associated with the development of NOAF both as continuous (OR = 0.86; CI 95% = 0.74–0.98, *p* = 0.02), and nominal variable (PNI < 41.5, OR = 1.87; CI 95% = 1.21–2.89, *p* = 0.004).

## 4. Discussion

In this cohort of postoperative cardiac surgery patients undergoing aortic valve replacement, we confirm that new-onset atrial fibrillation remains a common complication after cardiac surgery. In particular, in this population without a prior history of atrial fibrillation, we found that older age and lower PNI values emerged as independent predictors of NOAF also after adjustments for several established NOAF determinants as left atrial volume indexed, ejection fraction, surgical complexity and coronary artery bypass graft concomitant. Interestingly, among younger patients, PNI was the only factor significantly associated with AF onset. These findings contribute to the understanding of NOAF pathophysiology and support the role of individualized, targeted interventions in perioperative management.

Age has long been recognized as a major determinant of postoperative AF, reflecting the cumulative effects of atrial structural and electrical remodeling [44]. Indeed, previous studies have identified older age and left atrial enlargement as independent predictors of prolonged AF after SAVR [9]. In our cohort, only older age retained significance, while other established NOAF determinants as coronary artery bypass grafting, male gender, increased left atrial size, mitral valve disease, BMI were not associated with atrial fibrillation. Regarding the role of inflammatory markers in predicting NOAF following cardiac surgery, our results do not confirm a potential association between inflammation and new-onset atrial fibrillation.

Malnutrition has been widely acknowledged as a determinant of adverse postoperative outcomes. Perioperative malnutrition is associated with increased morbidity, mortality, hospital readmission, and longer length of stay [45,46]. In patients with aortic valve disease, poor nutritional status has been linked to higher all-cause mortality [47,48] and may represent a modifiable preoperative target. Despite evidence from the National Surgical Quality Improvement Program (NSQIP) highlighting malnutrition as a modifiable risk factor for poor surgical outcomes, the implementation of perioperative nutritional optimization remains limited [49]. Recent data further emphasize the importance of nutritional optimization as an effective perioperative strategy to reduce complications and improve recovery [50].

The Prognostic Nutritional Index, calculated from serum albumin and lymphocyte count, integrates both nutritional and inflammatory states. It has been established as a prognostic biomarker in various settings, including malignancies [36,37,51] and cardiac surgery. Lower PNI values have been independently associated with mortality and major cardiovascular events in patients undergoing coronary artery bypass grafting [52] and in valve surgery [53]. Similar findings have been reported in patients with severe aortic stenosis undergoing transcatheter or surgical AVR, where reduced PNI predicted increased mortality, morbidity, and prolonged ICU stay [54,55,56].

The interplay between malnutrition and inflammation is central to the development of postoperative complications, including AF. Chronic inflammation promotes atrial remodeling, while malnutrition exacerbates inflammatory responses and impairs immune defense, forming a detrimental cycle [57]. Serum albumin, one of the PNI components, exerts anti-inflammatory and antioxidant effects [58,59]; thus, hypoalbuminemia, reflecting both inflammation and inadequate protein intake, may predispose to oxidative stress–induced atrial injury [60]. Indeed, meta-analysis evidence indicates that low serum albumin is associated with increased AF risk [58].

Moreover, lymphocyte count, another PNI parameter, provides an index of immune competence. Reduced lymphocyte levels reflect impaired immune regulation, commonly observed in inflammatory conditions, and emerging evidence suggests that certain lymphocyte subsets exert protective roles against AF development [61]. These data support the concept that PNI captures both inflammatory and immune dimensions relevant to AF pathogenesis.

Previous studies have also demonstrated the predictive value of PNI for NOAF in patients with ST-elevation myocardial infarction undergoing percutaneous coronary intervention [38,39,40] and after coronary artery bypass grafting [62]. However, our study is the first to demonstrate an association between PNI and NOAF in patients undergoing surgical AVR. Notably, the predictive role of PNI was particularly pronounced among patients younger than 70 years, suggesting that nutritional and inflammatory imbalances may have a stronger relative impact when age-related structural remodeling is less prominent.

From a clinical standpoint, these findings further reinforce the importance of incorporating a comprehensive pre-operative nutritional assessment into existing risk stratification models for patients undergoing cardiac surgery. Adequate evaluation of nutritional and inflammatory status before surgery may provide additional prognostic information beyond traditional clinical and procedural risk factors. Unlike other nutritional indices that require complex calculations or specialized assessments, the PNI is derived from routinely available laboratory parameters, making it a simple, inexpensive, and easily reproducible tool for daily clinical practice. Its accessibility facilitates widespread implementation and supports its potential role in identifying patients at higher risk for adverse post-operative outcomes.

Given that new-onset atrial fibrillation has been consistently associated with prolonged hospitalization, increased rates of re-admission, and higher short- and long-term morbidity and mortality [63,64,65,66], the early identification of patients at increased risk is of paramount clinical relevance. In this context, pre-operative PNI screening may help clinicians recognize vulnerable individuals at an early stage, allowing for the adoption of targeted preventive strategies. These may include optimization of nutritional status, closer perioperative monitoring, and personalized perioperative management aimed at mitigating the risk of NOAF and its related complications.

The physiology of aging, which is commonly characterized by reduced nutrient intake, alterations in protein metabolism, sarcopenia, and a heightened pro-inflammatory state [67], further underscores the relevance of assessing nutritional status, particularly in elderly populations undergoing major cardiac surgery. Age-related immunological and metabolic changes may exacerbate susceptibility to post-operative arrhythmias and other complications. Nonetheless, the strong predictive role of PNI observed in younger patients in the present study suggests that subclinical nutritional and inflammatory impairments may also play a significant role in this subgroup. These findings indicate that nutritional screening should not be limited to older or frail individuals but should instead be systematically implemented in all patients undergoing major cardiac surgery, regardless of age, to improve risk stratification and support more tailored perioperative care strategies.

### Limitations

This study has several limitations that should be carefully considered when interpreting the results. First, this was a single-center investigation conducted on a relatively small sample size, which may limit the external validity and generalizability of the findings to broader or more heterogeneous populations. Moreover, the observational design of this study does not allow for the establishment of causal relationships between the prognostic nutritional index and the development of new-onset atrial fibrillation.

Second, variations in PNI during hospitalization were not evaluated, preventing an assessment of potential dynamic changes in nutritional and inflammatory status over time. In addition, detailed information regarding patients’ baseline nutritional habits, dietary intake, and long-term nutritional status prior to admission was unavailable, which may have influenced PNI values and introduced unmeasured confounding factors.

Third, some impactful operative variables as CPB time/cross-clamp time on development of NOAF were not available. However, as demonstrated in the novel logistic regression analysis, factors like the surgical complexity and CABG concomitance are not correlated with the developed of NOAF.

Despite these limitations, the present findings provide relevant clinical insights. Notably, PNI emerged as the only variable independently associated with NOAF after multivariable adjustment, highlighting its potential role as a meaningful marker in this clinical setting. This association was particularly evident among younger patients, suggesting that nutritional and inflammatory status may play a more pronounced role in the development of NOAF in this subgroup. Overall, these results support the potential utility of PNI as a simple and readily available tool for risk stratification while underscoring the need for larger, multicenter prospective studies to confirm these findings and further elucidate the underlying mechanisms.

## 5. Conclusions

NOAF is one of the most frequent complications after cardiac surgery, contributing to prolonged ICU stays, higher treatment costs, and increased morbidity and mortality. Our findings highlight preoperative nutritional assessment as an integral component of risk stratification and as a potential modifiable factor influencing postoperative outcomes. Incorporating nutritional optimization into perioperative care may help reduce complications and improve prognosis. Individualized approaches remain essential due to the multifactorial nature of nutritional and inflammatory states. In our study cohort, no deaths occurred during the hospitalization period; therefore, it was not possible to evaluate any possible association between NOAF and future adverse events or mortality. Future longitudinal studies would be of particular interest to assess whether the development of NOAF predicts long-term outcomes in this population. Specifically, investigating the relationship between NOAF and mortality or adverse events at 1-year, 2-year, and 5-year follow-up could help clarify the prognostic significance of NOAF beyond the acute phase.

## Figures and Tables

**Table 1:** The general baseline characteristics.

Characteristics	Cohort (n = 241)
Age [years] mean ± SD	66.2 ± 11.5
Male (%)	63.9
BMI (kg/m^2^) ± SD	27.1 ± 4.5
Smoking (%)	14.5
Hypertension (%)	65.1
Dyslipidemia (%)	47.7
Diabetes mellitus (%)	17
Hospital stay, day, mean ± SD	10.1 ± 4.3
Valve disease	
* Aortic stenosis (%)*	65.1
* Aortic regurgitation (%)*	34.9
* Bicuspid valve (%)*	42.3
***Therapy***	
* Anti-hyperlipidemic (%)*	43.6
* Anti-hypertensive (%)*	72.2
* Antiplatelet (%)*	34.9
* Anticoagulant (%)*	8.7
* Anxiolytic/Antidepressant*	12
* Antidiabetic drugs*	17
***Type of surgery***	
* AVR (%)*	57.3
* AVR and CABG (%)*	7.1
* AVR and Aorta surgery (%)*	21.2
* Aortic, mitral valve surgery (%)*	5
* Aortic, mitral valve and aorta surgery (%)*	2.1
* AVR and LAAC (%)*	1.2
* AVR, Aorta, LAAC*	2.5
* AVR, Aorta, CABG*	0.4
* Aortic, mitral valve and LAAC*	1.7
* Aortic, mitral valve and CABG*	1.2
***Surgical incision***	
* Full sternotomy (%)*	28.6
* Mini-sternotomy (%)*	51.5
* Mini-thoracotomies (%)*	19.9

BMI: body mass index; AVR: aortic valve replacement; LAAC: left atrial appendage closure; CABG: coronary artery bypass graft.

**Table 2:** Clinical, echocardiographic and bio-humoral characteristics of patients with and without postoperative AF.

Characteristics	NOAF Group (n = 84)	No NOAF Group (n = 157)	*p* Value
*Age [years] mean ± SD*	**68.9 ± 8.4**	**64.8 ± 12.6**	**0.007**
*Male (%)*	64.3	63.7	ns
*BMI (kg/m^2^) ± SD*	27.3 ± 4.2	27.1 ± 4.6	ns
*Smoking (%)*	14.6	14.3	ns
*Hypertension (%)*	72.6	61.1	ns
*Dyslipidemia* *(%)*	48.8	47.1	ns
*Diabetes mellitus (%)*	20.2	15.3	ns
*Hospital stay (day), mean ± SD*	**11.1 ± 4.7**	**9.5 ± 4.1**	**0.005**
**Valve disease**			
*Aortic stenosis (%)*	66.7	64.3	ns
*Aortic regurgitation (%)*	33.3	35.7	ns
**Type of valve**			
*Bicuspid valve (%)*	40.5	42.7	ns
*Tricuspid valve (%)*	59.5	57.3	ns
**Therapy**			
*Anti-hyperlipidemic (%)*	45.2	43.5	ns
*Anti-hypertensive (%)*	75	72.1	ns
*Antiplatelet (%)*	38.8	40.2	ns
*Anticoagulant (%)*	7.1	9.5	ns
*Antidiabetic drugs(%)*	20	16	ns
**Type of surgery**			
*AVR (%)*	56	58	ns
*AVR and CABG (%)*	6	7.6	ns
*AVR and Aorta surgery (%)*	22.6	20.4	ns
*Aortic, mitral valve surgery (%)*	7.1	3.8	ns
*Aortic, mitral valve and aorta surgery (%)*	2.4	1.9	ns
*AVR and LAAC (%)*	0	1.9	ns
*AVR, Aorta, LAAC*	1.2	3.2	ns
*AVR, Aorta, CABG*	0	0.6	ns
*Aortic, mitral valve and LAAC*	1.2	1.9	ns
*Aortic, mitral valve and CABG*	3.6	0	ns
**Surgical incision**			
*Full sternotomy (%)*	29.8	28	ns
*Mini-sternotomy (%)*	50	52.2	ns
*Mini-thoracotomies (%)*	20.2	19.8	ns
***Echocardiographic parameters***			
*Aorta*	39.5 ± 9.3	38.2 ± 7.8	ns
*Root*	35.4 ± 5.9	35.9 ± 6.8	ns
*Left Atrium Volume mean ± SD*	40.2 ± 15.9	38.4 ± 17.8	ns
*Left atrial volume indexed (LAVi)*	20.2 ± 9.6	18.3 ± 12.3	ns
*Left Atrium Area*	22.6 ± 6.2	23 ± 6.2	ns
*Left ventricular end-systolic dimension (LVESD)*	52.2 ± 8.1	52.8 ± 11.7	ns
*Left ventricular end-systolic volume (LVESV)*	139.8 ± 56.4	131.1 ± 53.9	ns
*Ejection fraction*	60.5 ± 8	60.6 ± 8.3	ns
*Peak velocity*	3.7 ± 1.2	3.5 ± 1.2	ns
*Mean gradient*	41.3 ± 21	41 ± 21	ns
*Posterior wall thickness*	10.7 ± 1.8	10.7 ± 8.1	ns
*Interventricular septum thickness*	12.6 ± 2.3	12.1 ± 2.1	ns
***Laboratory clinical-chemistry data***			
*Haemoglobin (Hb), g/dL*	13.9 ± 1.6	13.8 ± 1.4	ns
*Erythrocytes ×10^6^/μL*	4.7 ± 0.5	4.7 ± 0.5	ns
*Neutrophils, ×10^3^/μL*	4.0 ± 1.4	4.1 ± 1.6	ns
*Lymphocytes, ×10^3^/μL*	1.8 ± 0.5	1.8 ± 0.6	ns
*Monocytes, ×10^3^/μL*	0.65 ± 0.85	0.56 ± 0.16	ns
*Platelets, ×10^3^/μL*	219 ± 66	223 ± 60	ns
*C-reactive protein (CRP), mg/dL*	0.2 ± 0.2	0.3 ± 0.4	ns
*Fibrinogen, mg/dL*	353 ± 90	352 ± 97	ns
*Total cholesterol, mg/dL*	168 ± 37	174 ± 39	ns
*Low Density Lipoprotein Cholesterol (LDL), mg/dL*	96.4 ± 33	100 ± 35	ns
*High-Density Lipoprotein cholesterol (HDL), mg/dL*	54.4 ± 15	54 ± 15	ns
*Triglycerides, mg/dL*	93 ± 40	100 ± 49	ns
*Lipoprotein(a), nmol//L*	57.4 ± 72	61.4 ± 73	ns
*Creatinine, mg/dL*	1 ± 0.7	0.9 ± 0.2	ns
*Creatine phosphokinase (CPK), IU/L*	97 ± 61	113 ± 81	ns
*Alanine aminotransferase (ALT), IU/L*	19.8 ± 11	21 ± 16	ns
*Gamma-Glutamyl Transferase (GGT), IU/L*	37 ± 56.5	38 ± 75	ns
*Thyroid-Stimulating Hormone (TSH), mU/L*	3.5 ± 10.5	7.8 ± 62	ns
*Albumin, g/dL*	**4.2 ± 0.2**	**4.4 ± 0.3**	**0.01**
*Urea, mg/dL*	40.4 ± 14.2	41 ± 13.2	ns
*Glucose, mg/dL*	100.7 ± 19.2	100.8 ± 17.9	ns
*Neutrophil-to-lymphocyte ratio (NLR)*	1.9 ± 1.06	2.5 ± 1.3	ns
*Systemic immune inflammation index (SII)*	495 ± 367	573 ± 340	ns
*Systemic Inflammatory Response index (SIRI)*	0.34 ± 0.16	0.37 ± 0.34	ns
*Inflammatory prognosis index (IPI)*	0.09 ± 0.1	0.2 ± 0.3	ns
*Prognostic nutritional index (PNI)*	**40.7 ± 8.6**	**43.0 ± 2.4**	**0.01**
*Blood urea nitrogen to albumin ratio (BAR)*	9.1 ± 4.6	9.8 ± 3.4	ns

BMI: body mass index; AVR: aortic valve replacement; LAAC: left atrial appendage closure; CABG: coronary artery bypass graft; NOAF: New-onset postoperative atrial fibrillation.

**Table 3:** Multivariate logistic regression analyses showing the independent risk factors for NOAF after aortic valve replacement.

Variables	OR	95% CI	*p*-Value
*Age*	1.02	1.01–1.06	0.05
***PNI***	**0.9**	**0.8–0.9**	**0.** **03**
*Indexed left atrial volume*	1.0	0.9–1.03	0.63
*EF %*	0.9	0.9–1.03	0.76
*AVR vs. AVR + other surgeries*	1.05	0.56–1.95	0.87
*CABG concomitance*	1.07	0.38–2.98	0.89

AVR: Aortic valve replacement, PNI: prognostic nutritional index, EF: ejection fraction, CABG: coronary artery bypass graft.

## Data Availability

The data presented in this study will be made available by the authors on request.
